# Daily two-photon neuronal population imaging with targeted single-cell electrophysiology and subcellular imaging in auditory cortex of behaving mice

**DOI:** 10.3389/fncel.2023.1142267

**Published:** 2023-03-03

**Authors:** Junjie Huang, Susu Liang, Longhui Li, Xingyi Li, Xiang Liao, Qianshuo Hu, Chunqing Zhang, Hongbo Jia, Xiaowei Chen, Meng Wang, Ruijie Li

**Affiliations:** ^1^Center for Neurointelligence, School of Medicine, Chongqing University, Chongqing, China; ^2^School of Artificial Intelligence, Chongqing University of Technology, Chongqing, China; ^3^Brain Research Center and State Key Laboratory of Trauma, Burns, and Combined Injury, Third Military Medical University, Chongqing, China; ^4^School of Physical Science and Technology, Advanced Institute for Brain and Intelligence, Guangxi University, Nanning, China; ^5^Brain Research Instrument Innovation Center, Suzhou Institute of Biomedical Engineering and Technology, Chinese Academy of Sciences, Suzhou, China; ^6^Leibniz Institute for Neurobiology, Magdeburg, Germany; ^7^Institute of Neuroscience and the SyNergy Cluster, Technical University Munich, Munich, Germany; ^8^Guangyang Bay Laboratory, Chongqing Institute for Brain and Intelligence, Chongqing, China

**Keywords:** daily two-photon Ca^2+^ imaging, auditory cortex, behaving mouse, dendritic spines, loose-patch recording

## Abstract

Quantitative and mechanistic understanding of learning and long-term memory at the level of single neurons in living brains require highly demanding techniques. A specific need is to precisely label one cell whose firing output property is pinpointed amidst a functionally characterized large population of neurons through the learning process and then investigate the distribution and properties of dendritic inputs. Here, we disseminate an integrated method of daily two-photon neuronal population Ca^2+^ imaging through an auditory associative learning course, followed by targeted single-cell loose-patch recording and electroporation of plasmid for enhanced chronic Ca^2+^ imaging of dendritic spines in the targeted cell. Our method provides a unique solution to the demand, opening a solid path toward the hard-cores of how learning and long-term memory are physiologically carried out at the level of single neurons and synapses.

## Introduction

A central goal of neuroscience is to decipher the neural mechanism of the cerebral cortex during complex brain functions such as perception, learning and behavior. Long-term monitoring of neural dynamics in a limited set of neurons at different scales can facilitate investigations of these issues (Li et al., 2018; Tournissac et al., 2022). Two-photon imaging techniques have been widely used to examine various regions across the mouse cortex, providing a window to observe neurons at population and subcellular scales, such as dendrites and spines (Beaulieu-Laroche et al., 2019). Similarly, genetically encoded indicators (GECIs) are also acommonly applied and powerful tool for functional imaging that enables the monitoring Ca^2+^ activity of neurons (Chen et al., 2013; Zhang et al., 2021). Both methods are well-established and extensively used for acute or chronic observations in studies of different brain regions to explore the relationship between neuronal activity and behavior in awake mice. To date, chronic two-photon imaging in behavioral animals has focused mainly on the parietal brain region, including visual cortex (Garg et al., 2019; Yildirim et al., 2019), motor cortex (Prsa et al., 2017; Hwang et al., 2019), somatosensory cortex (Kim et al., 2012), cerebellum (Roome and Kuhn, 2020; Wagner et al., 2021; Redman et al., 2022; Yeon et al., 2022) and olfactory bulb (Tiret et al., 2009). With the popularization and subsequent advances in lateral brain imaging technology, chronic imaging of auditory cortex (AuC) has also shown steady progress (Francis et al., 2018; Li et al., 2018; Xin et al., 2019). Resnik and Polley used chronic two-photon Ca^2+^ imaging to track daily changes in layer 2/3 AuC neurons of mice with cochlear neurodegeneration (Resnik and Polley, 2021). However, real-time imaging to monitor neuronal changes and single-cell electrophysiology in the AuC during behavior training remains fraught with several challenges.

One fundamental issue in neuroscience is the exploration of how a single neuron integrates its receiving synaptic input and firing its spike output in the learning state, which would thus improve our understanding of the fundamental laws of neural circuit computation in the brain *in vivo*. In terms of the smallest unit of memory storage, pioneering researchers developed the “Synaptic-centric view of learning” (Gallistel and Matzel, 2013; Poo et al., 2016) and “Neuro-centric view of learning” (Titley et al., 2017) theories, respectively. In order to study the influence of the changes in dendritic spines and neurons on behavior, a particular need was put forward. Through the learning process, the neurons related to learning are tracked and recorded. Firstly, their output or firing characteristics were studied, and then the distribution and characteristics of their dendritic input were studied. However, supporting evidence in these seminal studies was primarily based on experimental results obtained in anesthetized animals or acute brain slices. It is, therefore, necessary to reproduce these experiments in a state of learning behavior.

To overcome the technical difficulty of patch-clamp recordings of targeted neurons in behaving animals, the input synaptic afferent signals and the somatic output electrophysiological recordings can be conducted separately. For single-cell electrophysiology, Huang et al. (2021) analyzed the relationship between Ca^2+^ and electrical activity in the visual cortex of GCaMP6 transgenic mice. Interestingly, researchers in several other studies designed automated patching systems combined with two-photon imaging to perform clamp recordings of cell bodies or even dendrites *in vivo* (Annecchino et al., 2017; Suk et al., 2017). For synaptic level imaging, the functional and morphological signal changes can be monitored after electroporation with organic dyes or GECI plasmids (Judkewitz et al., 2009; Broussard et al., 2018). However, despite these innovations, there is still no protocol for long-term subcellar imaging and electrophysiological recordings of target neurons after chronic two-photon imaging during the learning process. Several pioneers skilled in chronic cranial window imaging have provided us with ideas to solve this problem. Goldey et al. (2014) provided a protocol for window removal and replacement in the weeks and months after the initial craniotomy, which facilitated the further manipulations of the imaged cortex. Interestingly, two individual groups came up with the technique of long-term stable two-photon imaging in awake macaque monkeys (Sadakane et al., 2015; Li et al., 2017). Moreover, Roome and Kuhn developed a unique way of the chronic cranial window with an access port, which achieved long-term imaging and cellular manipulations in barrel cortex (Roome and Kuhn, 2014).

In order to decipher the dynamics of behavior-associated information, it is essential to precisely track long-term changes in specific neurons throughout behavior (Wang et al., 2020). Based on our previous studies and multiple pioneers’ protocols, we present an integrated method to decipher the neural dynamics during behavior at the population and subcellular levels. First, we improved a behavioral device for tilting the mouse head for fixation. Second, we conducted daily chronic imaging of the AuC in mice during behavior training. Third, the electrophysiological features of tracked neurons were verified after daily two-photon chronic imaging tracing. Finally, we demonstrate chronic subcellular imaging after daily two-photon chronic image tracing.

## Materials and methods

### Animals

Wild-type male mice (C57BL/6J) were provided by the Laboratory Animal Center at the Third Military Medical University. Before the chronic two-photon imaging experiments, the mice were housed in a 12 h-light/12 h-dark cycle (lights on at 7:00 am and lights off at 19:00 pm.) and provided with *ad libitum* access to food and water.

### Virus injection

Adult mice were anesthetized using 0.8–1.5% isoflurane in pure oxygen (0.5 L/min) and placed in a stereotaxic apparatus, with a feedback-controlled heating blanket to ensure that the internal body temperature was maintained at 37.5°C. The scalp was removed using scissors, and the eyes were covered by ointment (Bepanthen, Bayer, Germany). A circular craniotomy (∼0.5 mm diameter) was made at the injection site (AuC, AP (anteroposterior): −3.0 mm, ML (mediolateral): 3.8 mm). The virus (pAAV2/9-hSyn-GCaMP6m-XC) was loaded into a miniature glass pipette with a tip diameter of 20–30 μm. The pipette is slowly inserted at an angle below the surface of the AuC (angle: 70° to the horizontal plane), moving forward 1.4 mm, and injecting the virus (20 nl per min). 80 nl of the virus was injected into the target area and the pipette was hold-on in the target area for at least 5 min after injection to prevent backflow. After slowly retracting the pipettes, the cranial window is filled with bone wax and the scalp incision is closed with tissue adhesive (Vetbond, 3M Animal Care Products, USA). Animals were allowed to recover for at least 2 weeks until the virus was expressed.

### Implantation of the head-post

Dexamethasone (5 mg/kg) was injected subcutaneously into mice at least 2 h prior to surgery to prevent edema and inflammation. Mice were mildly anesthetized as mentioned in the previous section, followed by removing hair with hair removal cream and sterilizing the scalp with alcohol. The hemisphere and fascia of the skull were fully removed by scissors (Take extra care when cutting near the eyes and ears to prevent delayed healing or resulting in disruption of vision or hearing due to disruption of normal skin tone and/or severing of any nerves or blood vessels in these sensory organs). The titanium head-post was adhered to the skull surface by dental cement (Superbond, Sun Medical Co., Ltd). The adhering region of the dental cement and the skull was limited, that its anterior edge was <3.0 mm from the bregma and the right lateral edge was <3.5 mm from the midline. After 20 min of the head-post solidification, the long handle of the head-post was connected the head-post holder with secured by two screws.

### Implantation of chronic cranial window

The chronic cranial window implantation was performed after a 6 days recovery from the head-post implantation. The procedure of anesthesia and temporal hair removal in mice was the same as above. The temporal muscles and skin over AuC were removed and the temporal skull was etched with 2% H_2_O_2_. It was important to note that the temporal muscle should be carefully clipped along the temporal bone suture to prevent bleeding caused by damage to the surrounding blood vessels. The temporal skull over AuC was marked with a marker, and dental cement (Superbond, Sun Medical Co., Ltd.) was applied to the temporal skull and the customed recording chamber. After 20 min consolidation of the dental cement, a circular craniotomy (∼1.6 mm in diameter) was performed and the skull around the cranial window was polished to fit the coverslip optimally. It was important to avoid drilling in the same area for more than 2 s to prevent potential bleeding below the skull caused by overheating and vibration. The exposed AuC was submerged in artificial cerebral spinal fluid (ACSF) after being carefully cleaned with tweezers. The cranial window consisted of a minor coverslip (1.5 mm in diameter) and a larger one (2.5 mm in diameter). The interspaces of these two coverslips were sealed with ultraviolet-cured optical adhesives (Norland Products Inc., USA). The edges of the coverslips were sealed with N-flow dental acrylic (Ivoclar, Liechtenstein). Note that, during the sealing time, the temporary fixation was performed by a slight pressure application of the fashioned cotton-tipped applicator above the cranial window (which remains submerged in ACSF). Double-check the tightness of the chronic cranial window and the potential bleeding points under the coverslips.

In the end, the mice were given subcutaneous injections of cefazolin for 3 days (500 mg/kg, once per day, North China Pharmaceutical Group Corporation, China). The chronic imaging was performed at least 7 days recovery to prevent inflammation.

### Replacement of chronic cranial window

The removal of chronic cranial window was performed after verification of the targeted cell by daily chronic two-photon imaging. The procedure of anesthesia in mice was the same as described above, and then the mice were fixed to head-post holder. The recording chamber was filled with 70% (vol/vol) ethanol for at least 90 s. The dental cement around the cranial window was drilled carefully until exposure of the underlying skull, and any debris caused by drilling was cleared by ACSF. The rotation speed of skull drill should be reduced for minimizing the vibration of cranial window. The central part of the window should remain snug to the cortex during the removal stage of cranial window, with the tips of forceps closed and gently placed above the cranial window. In this way, it can minimize the risk of popping out, brain damage or bleeding during the craniotomy. The recording chamber was flushed incessantly by ACSF for removing any debris. After confirming that there was no bleeding, inflammation and debris over the cortex, the cranial window was gently removed and immediately irrigated with ACSF. The reimplantation of the chronic cranial window was performed after single-cell electroporation and the procedure was the same as above.

### Sound stimulation

Two different chords were chosen as sound stimuli in the experiment (Wang et al., 2020). Chord 1 consisted of 2.0, 2.7, 3.6, and 4.9 kHz, while chord 2 consisted of 8.9, 12.1, 16.3, and 22.0 kHz. Chord 1 has a sound level of around 78 dB SPL, and chord 2 has a sound level of around 71 dB SPL. The sound level of the background noise (∼55 dB SPL) is lower than these two chords. In our previous studies, it was proposed that background noise is mainly composed of low frequencies (<1 kHz). The peak of the background noise is below 1 kHz and the spectral density is ∼33 dB/sqrt (Hz). The duration of a sound stimulus (chord 1 or chord 2) was 50 ms. An ED1 electrostatic speaker driver and a free-field ES1 speaker (both from Tucker Davis Technologies) together produce the sound stimulus. The mouse ear (contralateral to the imaged AuC) was positioned 6 cm away from the speaker. The sound stimuli were custom written using a LabVIEW-based program (LabVIEW 2015, National Instruments) and converted to analog voltages using a PCI6251 card (sampling at 1 MHz, National Instruments).

### Training procedure

After implanting the head-post, the mice were sent back to their home cages for a 3 days recovery. Then the mice were handled for more than 10 min per day for 3–4 days. At the same time, the head fixation was put in their home cages for habituation. A few mice were unadopted the fixation apparatus would be eliminated, which showed depression or loss >20% of original body weight. The remaining mice that can adapt to the head fixation would be performed chronic window implantation. It takes about 7 days for the mice to recover and adapt to chronic window after chronic window implantation. During this period, ∼10% of mice were eliminated due to the inflammation of the cranial window. Behavior training continued until the mice were habituated to the cranial window and the fixation apparatus.

A sound-triggered licking task was designed with simultaneous two-photon imaging conducted during the training. Each trial consisted of a sound stimulation period (50 ms), an interval (100 ms) between stimulation and reward, and a water delivery period (20 ms). The sound stimulus used either chord 1 (kHz: 2.0, 2.7, 3.6, and 4.9) or chord 2 (kHz: 8.9, 12.1, 16.3, and 22.0), and inter-trial intervals (ITI) were set in the range of 4–10 s. In the training, each sound stimulus corresponded to the lick-port in the given position, with chord 1 corresponding to the left lick-port and chord 2 corresponding to the right lick-port. To avoid compulsive licking, the distance between the lick-port and the lower lip was adjusted to 4 mm, so the mouse could just touch the lick-port at a distance. A ∼4 μl drop of water was delivered 150 ms after the onset of a sound stimulus. Approximately 300 trials of a training day were divided into 6 sessions, with each trial session followed by a rest period of at least 5 min. Mice typically did not perform well in the first three training days, and ∼0.5 ml of supplementary water was provided to prevent dehydration since the water obtained from lick-ports was less than required. A weight loss (<20%) of mice was facilitated for training (Guo et al., 2014). Generally, the mice became expertly conditioned to the two chord-triggered licking tasks after 6 training days, which was defined as a success rate of >80% in the behavioral task. Each training trial was performed with simultaneous two-photon imaging, and the licking movements were monitored by an infrared camera (30 Hz scanning).

### Daily chronic two-photon imaging

Daily chronic two-photon imaging was performed during each behavioral training session to track changes in neural dynamics of the AuC. The head-fixed mice were placed under an upright two-photon microscope during the behavioral training. The same focal plane was imaged daily across the whole training stage. A custom-built two-photon microscope system based on using a 12.0 kHz resonant scanner (model “LotosScan 2.0,” Suzhou Institute of Biomedical Engineering and Technology) was used for two-photon imaging. A mode-locked Ti:sapphire laser (model “Mai-Tai DeepSee,” Spectra Physics, excitation wavelength: 920 nm) was used to excite calcium sensors. For all experiments, a 40 × /0.8 NA water-immersion objective (3.5 mm WD, Nikon) was used for imaging. The typical focal plane size for population scale imaging was 300 μm × 300 μm, and the focal plane size for subcellular imaging was 25 μm × 25 μm. Low laser illumination (10–40 mW laser power) was selected for optimal imaging, depending on the depth of FOV (Field of view).

### Loose-patch recordings after chronic two-photon imaging

Targeted loose-patch recordings after chronic two-photon imaging were performed in behaving mice as described in detail in our previous studies (Li et al., 2018; Xin et al., 2019). In brief, a glass pipette filled with ACSF and 100 μM OGB-1 6K^+^ (Molecular Probes) (5–8 MΩ) was applied with 30 mbar pressure to approach the neuron of interest. As the pipette moved to the center of the neuron, the positive pressure was released and a negative pressure of 50–100 mbar was applied until the tip resistance of the pipette reached 30 MΩ. Voltage clamp recording was carried out with an EPC10 amplifier (HEKA Elektronik, Germany). The electrophysiological data were filtered at 10 kHz and sampled at 20 kHz using Patchmaster software (HEKA Elektronik, Germany).

### Single-cell electroporation after chronic two-photon imaging

Similar to methods in previous study (Judkewitz et al., 2009), after daily two-photon chronic image tracing, dendrites and spines of the targeted neurons were labeled by electroporation. First, the relative distance and angle between the targeted neuron and the pipette were calculated. Then, a high resistance (12–15 MΩ) borosilicate pipette filled with plasmid (130 ng/μL syn-axon-GCaMP7b-WPRE-pA and 100 μM OGB-1 6K^+^) was mounted on a pipette holder connected to a micromanipulator (Luigs & Neumann, Germany) and moved to approach the targeted neuron. The plasmid was loaded into neurons by 100 rectangular pulses (−10 V, 0.5 ms duration, 50 Hz) triggered by the Master-8 (A.M.P.I, Israel) and delivered by the MVCS-01 iontophoresis system (NPI Electronic, USA). The same pipette could be used to electroporate fewer than 3 traced neurons.

### Quantification and statistical analysis

The custom-written software LabVIEW 2015 (National Instruments, USA), Prism 8 (GraphPad Software, USA), Igor Pro 6.0 (Wavemetrics Inc., USA), and MATLAB 2018b (MathWorks, USA) were used to analyze the data offline. A custom correction software (Matlab 2018b, Mathworks) was used for correcting motion-related artifacts of imaging, which was written based on the image plugin software TurboReg (ImageJ, NIH, USA). Frame-by-frame alignment for the imaging data was performed by translation algorithm, and the imaging data were registered to the average image of the first 100 frames of the FOV in each imaging day.

To assess the performance of mice on a chords-triggered licking task during learning, the success rate of the licking response was recorded by real-time video captured by an infrared camera. A 500 ms detection window was limited for calculating successfully licking. In detail, a region of interest (ROI) was manually drawn in the video frame between the lip of mice and the lick-port.

To extract the fluorescence signal, same as in previous studies (Li et al., 2018; Wang et al., 2020), the ROI based on the fluorescence intensity was performed mapping. The fluorescence changes (f) were calculated to correspond to pixel values in each specified ROI. Relative fluorescence changes (Δf/f) were used in most Ca^2+^ response data in this paper. The baseline fluorescence (f0) was estimated as the 25th percentile of the entire fluorescence recording and the relative fluorescence change Δf/f = (f-f0)/f0 was calculated as the Ca^2+^ signal.

To reconstruct the morphology of the neurons, the Z-stack fluorescent images of individual neurons were projected into an average image using projection software (Amira, USA). Based on the average projection, the morphology of neurons including soma and dendrites was manually drawn using Adobe Illustrator CS6 (Adobe Systems, USA). In order to determine statistical significance (*P* < 0.05), the comparison data from the psychometric test was used for a two-tailed *t*-test (paired). The comparison of Ca^2+^ signals of population neurons, spines, and active dendritic shafts used one-way ANOVA test.

## Results

### The updated head fixation apparatus and auditory associative learning task

**FIGURE 1:**
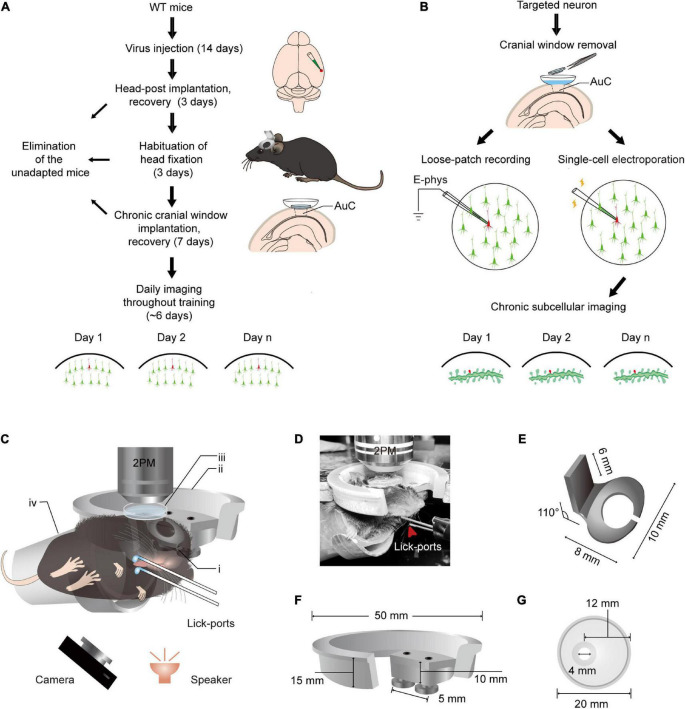
Training paradigm and updated head fixation apparatus for daily two-photon Ca^2+^ imaging in the AuC of behaving mice. **(A)** Technical flowchart for chronic population two-photon Ca^2+^ imaging across sound evoked licking behavior training. **(B)** Procedure of loose-patch recording and subcellular structure imaging of target neuron after population imaging tracing. **(C)** A diagram of the setup for simultaneous daily two-photon chronic imaging during training. The camera was used to monitor licking behavior in infrared mode and the speaker was placed 6 cm below the mouse’s left ear. (i) Head-post (panel **E**): (ii) recording holder (panel **F**); (iii) recording chamber (panel **G**); (iv) body tube; 2PM: Two-photon microscope. The detailed size and design of these parts are present below. For assembly of head-post, recording holder, and recording chamber, please see details in Figure 2. **(D)** An example mouse during training with a sound-evoked licking task with simultaneous two-photon imaging. **(E)** Titanium head-post. Lower part with curved notch for attachment to the skull by dental cement, and an upper holder for fixation at a 110° angle relative to the lower part. **(F)** Device for connecting the microscope to the head fixing post. Two screws are used in the device to provide a firm attachment to the head fixing post and thus reduce distortions in imaging caused by mouse movements. **(G)** Plastic recording chamber suitable for chronic imaging in the AuC of mice (upper view). The cranial window opening on one side of the chamber is more suitable for long-term wear in mice.

The flowchart for chronic two-photon imaging of mice during sound-triggered licking training is shown in Figure 1A. The preparation of the animal begins with virus injection, followed by implantation of the head-post and chronic cranial window. Some mice failed to adapt to the head rotation fixation procedure or developed inflammation after the chronic cranial window was implanted. Mice that were able to adapt began performing chord-triggered licking tasks with simultaneous chronic two-photon imaging. In addition, loose-patch recordings and electroporation of target neurons were performed, respectively (Figure 1B). The chronic subcellular imaging was performed over days after successful expression of plasmid. Figure 1C shows a schematic with the precise dimensions of the updated head fixation apparatus. Head-fixed mice were trained in a dark environment to associate the two chords and the different locations of the lick-ports (Figure 1D). Three improvements to the apparatus included: (1) the base of the head-post (Figure 1E) was curved to fit the skull and was mostly hollowed out for the application of bonding; (2) the head-post holder (Figure 1F) was changed from a normal slot to two screw fasteners, resulting in greater stability; (3) the opening of the recording chamber (Figure 1G) was decentered for long-term imaging and to allow unshielded eyes on the temporal side of the mice. According to the method of behavioral training (Supplementary Figure 1A), water was pumped (20 ms) at a constant delay (100 ms) after sound stimulation (50 ms). A droplet remained on the spout until voluntarily licked by the animal or replaced by a new drop in the next trial. There were no punishment or behavior-enforcing events during training, regardless of the behavior of the mice, each sound stimulus was followed by a water reward.

As meaningful sound stimuli are usually complex sounds in human beings or animals (Suri and Rothschild, 2022), complex sounds were used for unique stimuli. Chord 1 in our training method consisted of tones of 2.0, 2.7, 3.6, and 4.9 kHz, followed by water delivery at the left lick-port, while chord 2 consisted of tones of 8.9, 12.1, 16.3, and 22.0 kHz (Supplementary Figure 1A), followed by water delivery from the right lick-port. Most mice achieved stable conditioning in their performance (success rate > 80%) after 6 training sessions (Supplementary Figure 1B), meaning that an association between complex sounds and a corresponding lick-port was successfully established in these mice. Since body weight is also an important factor in the behavioral training of mice, mice that lost more than 20% of their body weight were necessarily excluded. Normalized values of individual body weights over the study period (Supplementary Figure 1C) showed a slight decrease and then remained stable (*N* = 6 mice). A psychometric behavior test using each constituent tone (Supplementary Figure 1D) showed that the probability each individual tone would evoke a licking response was significantly lower than that of the response to either chord stimuli.

### Stable functional imaging of populations across multiple days

**FIGURE 2:**
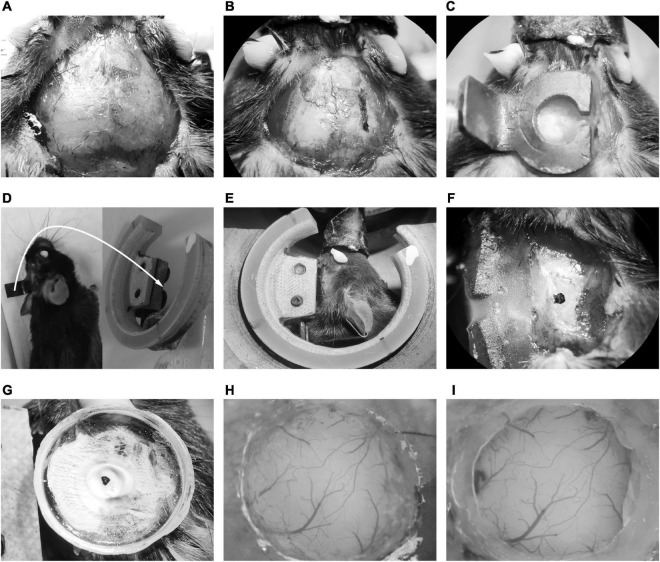
Illustration of chronic cranial window implantation. **(A)** Skin removal and tissue cleaning. **(B)** The lateral edge marking of the head-post. **(C)** Fixation of the head-post with dental cement. **(D)** The mice embedded with head-post (left) and the head-post holder (right). The white arrow indicated that the long handle of the head-post was connected to the slot of the recording holder with two screws. **(E)** The head fixing mice on the head-post holder. **(F)** Exposure of temporal skull. **(G)** Fixation of the recording chamber. **(H)** The craniotomy of AuC. **(I)** The cranial window embedding of AuC.

**FIGURE 3:**
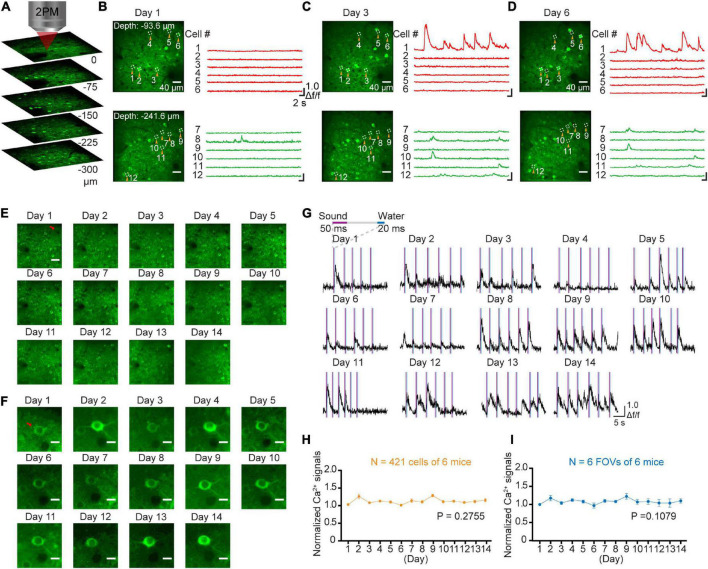
Stable long-term chronic two-photon imaging. **(A)** 3D reconstruction of population neurons labeled by the GcaMP6m-XC virus in AuC. **(B)** Two photon imaging in AuC at different depths on day 1. **(C)** Two photon imaging in the same FOVs (in panel **B**) on day 3. **(D)** Two photon imaging in the same FOVs (in panel **B**) on day 6. **(E)** Daily two-photon chronic imaging of AuC neurons labeled by GCaMP6m-XC virus across as long as 14 days. Scale bar: 40 μm. **(F)** Daily two-photon chronic imaging of an example magnified neuron out of panel **(E)** across 14 days. Scale bar: 5 μm. **(G)** Ca^2+^ signals of an example neuron by expressing GCaMP6m-XC virus across as long as 14 days. **(H)** Comparison of normalized Ca^2+^ signals of population neurons during chronic two-photon imaging. (421 cells from 6 mice), *P* = 0.2755, one-way ANOVA test. **(I)** Comparison of normalized Ca^2+^ signals of field of view during chronic two-photon imaging (6 FOVs from 6 mice). There was no significant difference in daily Ca^2+^ signals during imaging, *P* = 0.1079, one-way ANOVA test.

The GCaMP-XC virus was used to track cells because it reduces neuronal toxicity while improving calcium quality and reliability, thus allowing long-term, high expression (Geng et al., 2022). Chronic two-photon imaging was performed after the implantation of cranial window (Figure 2, see detail in the section “Materials and methods”). Side view images were reconstructed in different depths (Figure 3A). The example images at different depths were generated by averaging 600 frames and the Ca^2+^ signals of selected neurons during chronic two-photon imaging were shown in Figures 3B–D. The GCaMP6m-XC viral neurons could be tracked in the same imaging plane across 14 days (Figures 3E, F). The Ca^2+^ signals of the targeted neuron (Red arrow in Figure 3F) in response to sound stimuli were repeatedly imaged for 14 days (Figure 3G). Normalized Ca^2+^ signals of individual neurons (Figure 3H) from six different imaging planes (Figure 3I) were examined by chronic two-photon imaging (*N* = 6 mice).

### Loose-patch recording after chronic two-photon imaging

**FIGURE 4:**
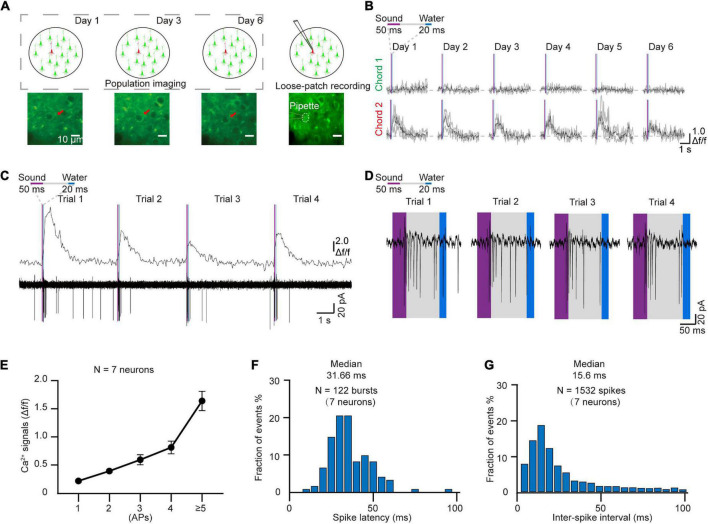
Loose-patch recording after daily two-photon chronic imaging. **(A)** Schematic diagram of loose-patch recording after daily two-photon imaging on the AuC. **(B)** Ca^2+^ signals responded to two chords during chronic two-photon imaging (Red arrow marked in panel **A**). **(C)** 4 Consecutive trials of Ca^2+^ signals and corresponding loose-patch responses of targeted neurons (Tracked by chronic imaging in panel **B**) in the AuC of the behaving mouse. **(D)** Magnified view of the action potential (AP) in panel **(C)**. **(E)** Correlation of the different numbers of action potentials and their corresponding amplitudes of Ca^2+^ transients (97 trials of 7 neurons from 5 mice). **(F)** Spike latency histogram for all the response events (122 burst events of 7 neurons). **(G)** Inter-spike interval histogram for all the response events (1532 spikes of 7 neurons).

In order to verify the electrophysiological features of the targeted neurons after chronic two-photon imaging, we next performed loose-patch recordings of the same targeted neurons (Kitamura et al., 2008; Beaulieu-Laroche et al., 2019). For these experiments, the coverslip was removed from the cranial window at the end of the behavior training sessions (Figure 4A). The Ca^2+^ signals of the tracked neurons in the AuC showed a high correlation with sound stimuli throughout the training task (Figure 4B). Two-photon Ca^2+^ imaging concurrent with loose-patch recording indicated that Ca^2+^ signals and corresponding electrical signals were both highly correlated with the sound stimuli (Figure 4C). At the same time, close examination of individual spike patterns showed that larger amplitude Ca^2+^ signals were accompanied by a short, intense release of electrical signals (Figure 4D). Statistical analysis indicated that Ca^2+^ signal amplitude was positively correlated with number of action potentials (Figure 4E, 7 neurons in 97 trials of 5 mice). The histogram of spike latency distribution (Figure 4F) was shown as approximately 32 ms (31.66 \25.44–40.64 ms, median \25%–75% percentiles) delay time after sound stimuli. The median of inter-spike interval was ∼16 ms (15.6 ms \9.8–28.77 ms), which corresponded to a high firing rate of 64.1 Hz (34.8–102 Hz) (Figure 4G).

### Long-term subcellular imaging of single electroporated neurons after daily two-photon somatic imaging

**FIGURE 5:**
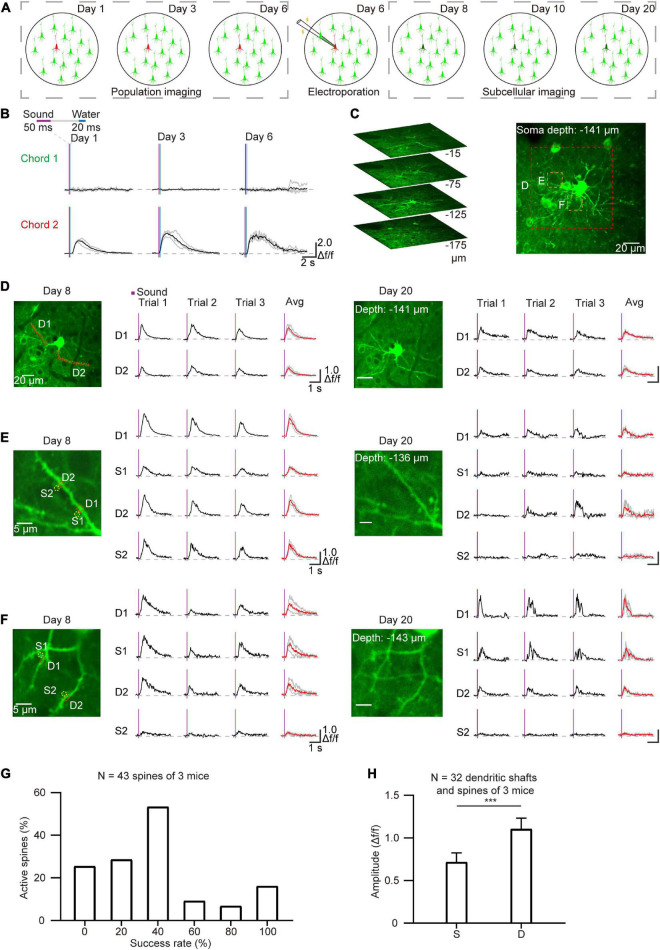
Chronic subcellular imaging after daily two-photon population imaging. **(A)** Schematic diagram of chronic subcellular imaging after daily two-photon somatic imaging on the AuC. Day 1–6: daily two-photon population imaging; Day 6: targeted electroporation after chronic imaging; Day 8–20: chronic subcellular imaging of the targeted neuron. **(B)** Ca^2+^ signals of the targeted neuron respond to two chords during chronic two-photon imaging. **(C)** Reconstruction of the targeted cell after electroporation. **(D)** Chronic subcellular two-photon imaging of the same dendrite segments after electroporation (red dashed region outlined of panel **(C)**, Day 8–20). **(E,F)** A similar arrangement as panel **(D)** showing Ca^2+^ signals of the dendrites and spines (yellow dashed region outlined of panel **C**). The red lines represent averaged signals from three consecutive trials, and the green lines represent independent signals. **(G)** Reliability of sound-evoked Ca^2+^ signals in active dendritic spines (*n* = 43 spines, 3 neurons, calculated from 6 consecutive trials). **(H)** Amplitude histogram of sound-evoked Ca^2+^ signal in active spines (S) and active dendritic shafts **(D)** (*n* = 32 dendritic shafts, 3 neurons), ***P = 0.0006, two-tailed *t*-test.

Single-cell electroporation is an effective method for labeling individual neurons with fluorescent dyes and/or plasmid DNA (Judkewitz et al., 2009; Han et al., 2018; Wang et al., 2022). In order to perform long-term imaging at the subcellular level, single cells were labeled by electroporation after daily population-scale two-photon imaging of AuC neurons that responded to sound stimuli (Figure 5A). After removing the cranial window (see section “Materials and methods”), neurons that displayed a strong response to sound stimuli in chronic two-photon imaging (Figure 5B) during the training process were electroporated with the GCaMP7b plasmid. Electroporated neurons were then performed chronic subcellular imaging. Subsequently, side-view images and Z-stack images of the example targeted neuron were reconstructed after electroporation (Figure 5C). The Ca^2+^ signals of the dendritic shafts and spines were also recorded on the second and fourteenth day after plasmid electroporation to detect response to chord stimuli in dendrites and spines (Figures 5D–F). The results verified that the GCaMP was expressed in neurons and that dendrites and spines were visible, and notably, the sound-evoked signals both in dendrites shafts and spines were highly related to the sound stimuli (Figures 5D–F). We then determined the stability and consistency of sound-evoked Ca^2+^ responses for each active spine in 5 consecutive trials (*n* = 43 spines, 3 neurons, Figure 5G). Statistical analysis of the mean amplitude of Ca^2+^ response revealed a significantly different between active spines (32 out of 43 spines) than that detected in their corresponding dendritic shafts (Figure 5H). These cumulative data indicated that this integrated approach to *in vivo* imaging in behaving mice could be used to stably capture the activity of brain regions or subcellular neuronal structures over the course of days or weeks.

## Discussion

In summary, the current study examines neuronal activity in the AuC of behavioral mice through daily two-photon imaging using the GCaMP6m-XC sensor, which exhibits long-term stability suitable for capturing changes in activity under prolonged stimuli or behavioral conditioning. To accommodate long-term observation of neural activities in AuC, we also developed an updated apparatus for head-fixation, and demonstrate its application in imaging the AuC of mice exhibiting behavior in response to auditory stimuli. This method offers three main advantages over previous approaches. First, *in vivo* imaging can be captured simultaneously with sound-evoked training for licking behavior. Second, this method enables new approaches for characterizing the electrophysiological signal patterns, as demonstrated by loose-patch recordings in long-term behaving mice after prior image-based cell tracking. Third, electroporation of GECI plasmids in this method allows long-term subcellular level imaging of tracked neurons even after conducting daily population scale imaging. The collective benefits of this method thus provide an effective means for interrogating long-term neural dynamics in targeted neurons at multiple scales in behaving mice.

The key to high-fidelity two-photon imaging in awake, behaving mice is to reduce motion in the brain during imaging (Dombeck et al., 2007; Greenberg and Kerr, 2009). In previous work, we developed a method for two-photon Ca^2+^ imaging with head fixation in head-rotated mice (Li et al., 2018). However, as the number of days for imaging increased, brain motions became larger, resulting in especially high variability at the subcellular level. Using an updated head-post in conjunction with the modified recording chamber in the current study, dendrites and spines of tracked could be imaged with high stability for up to 13 days.

For AuC research, two-photon imaging was historically conducted by tilting the objective to fit upright head-fixed mice. For instance, Kato et al. (2015) used a tilted objective to reveal the bidirectional modulation of sensory representation in AuC. In Kuchibhotla et al. (2017) also used this approach to demonstrate that parallel processing of cholinergic modulation by interneurons can enable context-dependent behavior. However, two-photon imaging of the AuC by tilting the objective does not allow further exploration of the AuC, for example by electroporation of secondary reporters or targeted loose-patch recordings. The rotating head strategy used in this study allows the horizontal recording chamber to be filled with a liquid medium, thus enabling a wider range of experiments than the previous strategy of tilting the objective to accommodate mice with upright head fixation. This innovation led to our further loose-patch recordings and single-cell electroporation of any neuron of interest during long-term two-photon imaging.

Targeted patch-clamp recording is a ubiquitous and powerful neuroscience research method for characterizing the electrophysiological properties of neurons in intact neural circuits. Suk and co-workers developed an “image patching” robot to characterize identified cell types in intact neural circuits (Suk et al., 2017). With loose-patch recordings of dendritic branch structure, Landau and co-workers revealed branch-specific variation in bAP-dependent Ca^2+^ signals (Landau et al., 2022). However, electrophysiological recordings in that study were performed in acute experiments and were thus blind to the former neural dynamics. Remarkably, Roome and Kuhn achieved electrophysiological recording after long-term imaging in barrel cortex by chronic cranial window with an access port (Roome and Kuhn, 2014). However, Chronic cranial window with access port was usually performed in somatosensory cortex and cerebellum, and chronic cranial window was covered by a 5 mm diameter coverslip, which was much larger than the narrow space of AuC with 1.5 mm in diameter. Due to the limited space in AuC, it was hard to approach the cell in the imaging area arbitrarily with the access port. Based on our method, long-term tracking of the electrophysiological properties of neurons in AuC can be performed by removing the coverslips.

For imaging at dendritic spine level, sparse labeling of neurons by viruses (Li et al., 2017) or electroporation permits the specific labeling of a limited number of cellular structures (Kitamura et al., 2008). In addition, new techniques, such as 3D DRIFT AO scanning, allow for more flexible and rapid imaging of complex subcellular structures (Szalay et al., 2016). However, these recordings are also largely captured through acute experiments and random neurons. The integrated method described here allows long-term imaging of dendritic spines tracked neurons to uncover precise changes in neural dynamics from the cell population to dendritic spine levels. Our method may therefore provide a powerful tool for investigating plasticity in single neurons in the near future.

In recent years, several powerful GECIs were developed for *in vivo* study of neural activities. For example, XCaMPs provide significantly enhanced spatial resolution (Inoue et al., 2019) while ‘jGCaMP8’ offers better temporal resolution (Zhang et al., 2021). It should be noted that XCaMPs offer multiple color channels for functional imaging, and each color can be linked to neuron subtype. Combining these indicators, our method allows long-term two-photon imaging of the deep cortex or other brain regions by Grin lens (Yang et al., 2019) while labeling neurons of interest with another color Ca^2+^ indicator for contrast with the labeled population. Combining these tools will result in better signal-to-noise ratios in recording different focal planes in future work. Moreover, advanced genetically encoded indicators of neurotransmitters and neuromodulators have been developed in recent years for *in vivo* monitoring of dynamic changes in neurotransmitters (Wu et al., 2022). By combining these indicators, dynamic changes in neurotransmitters can be monitored in individual dendritic spines, raising exciting possibilities for our method to examine the role of neurotransmitters in various physiological and pathological states. In addition, recent advances in two-photon optogenetics now allow the manipulation of a set of neurons by two-photon illumination, facilitating a broader scope investigation of the soma and dendritic regions of targeted neurons during multiple behaviors (Carrillo-Reid et al., 2019). In conclusion, this method provides an alternative strategy for deciphering the neural dynamics of target neurons during multiple behavior paradigms.

### Future prospect

Here, we present an integrated method of daily two-photon neuronal population Ca^2+^ imaging through an auditory associative learning course, followed by targeted single-cell loose-patch recording and electroporation of plasmid for enhanced chronic Ca^2+^ imaging of dendritic spines in the targeted cell. On one hand, the long-term skill training to perform visually guided patching and subcellular imaging in behaving mice limited widespread application of this method. The “image patching” robot can be introduced in future experiments (Annecchino et al., 2017; Suk et al., 2017). On the other hand, the same green color labeling of both soma and dendritic spines may lead to crosstalk of GCaMP6 and GCaMP7b sensor, even though the brightness of the dendritic spines was increasing in the initial days after electroporation. The combination of GCaMP indicator and RCaMP indicator will be facilitated for dual color imaging in soma and dendritic spines.

## Data availability statement

The raw data supporting the conclusions of this article will be made available by the authors, without undue reservation.

## Ethics statement

The animal study was reviewed and approved by the Third Military Medical University Animal Care and Use Committee.

## Author contributions

XC and RL designed the study. RL and JH performed all the *in vivo* experiments and data analysis with support from the other authors as follows. SL, MW, LL, and XLi contributed to the custom-built instruments and technical support. MW, HJ, and XC established the experimental methods. XLiao, XC, HJ, and QH established the data analysis methods. RL, HJ, XC, MW, and QH wrote the manuscript. All authors discussed and commented on this manuscript and contributed to the article and approved the submitted version.

## Supplementary material

Supplementary Figure 1 Training procedure of the sound-triggered licking task in water-restricted mice. **(A)** Paradigm diagram of daily training and two-photon imaging. Two chords were used during the training alternately (Chord 1: 2.0, 2.7, 3.6, and 4.9 kHz; Chord 2: 8.9, 12.1, 16.3, and 22.0 kHz). **(B)** Behavioral performance across sound evoked licking behavior training (*N* = 8 mice). **(C)** Normalized weight changes across 6 training days (*N* = 6 mice). The dashed line indicates 20% weight loss. **(D)** Psychometric tests in which 2-tone-trained mice were stimulated with either the two chords or individual constituent tones. Chord 1 versus its constituent tones, *P* = 0.0004, *P* = 6.7748e−06, *P* = 0.0045, *P* = 1.1874e−04, respectively, two-tailed *t*-test, *n* = 6 mice; chord 2 versus its constituent tones, respectively, *P* = 0.0082, *P* = 0.0037, *P* = 0.0033, *P* = 3.7597e−04, two-tailed *t*-test, *n* = 6 mice. Data with error bars are expressed as mean ± s.e.m. ****P* < 0.001, ***P* < 0.01, **P* < 0.05.
