# Supplementary figures and images for: Daily two-photon neuronal population imaging with targeted single-cell electrophysiology and subcellular imaging in auditory cortex of behaving mice
Source: Front Cell Neurosci. 2023 Mar 3;17:1142267. doi: 10.3389/fncel.2023.1142267 (PMC10020347; doi:10.3389/fncel.2023.1142267)

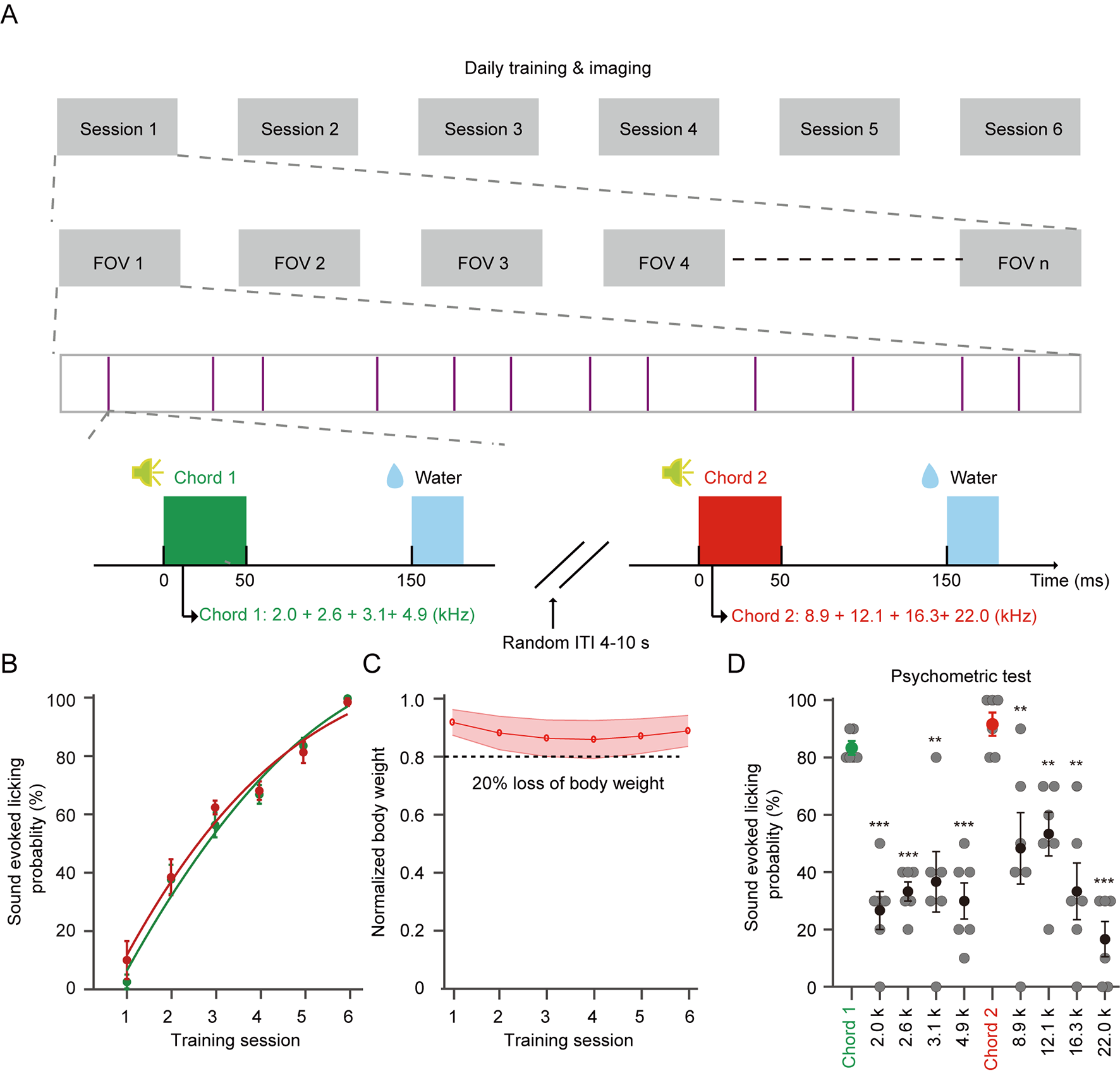

Supplement: Supplementary Figure 1 — Training procedure of the sound-triggered licking task in water-restricted mice. (A) Paradigm diagram of daily training and two-photon imaging. Two chords were used during the training alternately (Chord 1: 2.0, 2.7, 3.6, and 4.9 kHz; Chord 2: 8.9, 12.1, 16.3, and 22.0 kHz). (B) Behavioral performance across sound evoked licking behavior training (N = 8 mice). (C) Normalized weight changes across 6 training days (N = 6 mice). The dashed line indicates 20% weight loss. (D) Psychometric tests in which 2-tone-trained mice were stimulated with either the two chords or individual constituent tones. Chord 1 versus its constituent tones, P = 0.0004, P = 6.7748e−06, P = 0.0045, P = 1.1874e−04, respectively, two-tailed t-test, n = 6 mice; chord 2 versus its constituent tones, respectively, P = 0.0082, P = 0.0037, P = 0.0033, P = 3.7597e−04, two-tailed t-test, n = 6 mice. Data with error bars are expressed as mean ± s.e.m. ***P < 0.001, **P < 0.01, *P < 0.05. [file Image_1.TIF]
